# Implementation of multi-omics in diagnosis of pediatric rare diseases

**DOI:** 10.1038/s41390-024-03728-w

**Published:** 2024-11-19

**Authors:** Sara S. Ali, Qifei Li, Pankaj B. Agrawal

**Affiliations:** Division of Neonatology, Department of Pediatrics, University of Miami Miller School of Medicine and Holtz Children’s Hospital, Jackson Health System, Miami, FL USA

## Abstract

**Abstract:**

The rapid and accurate diagnosis of rare diseases is paramount in directing clinical management. In recent years, the integration of multi-omics approaches has emerged as a potential strategy to overcome diagnostic hurdles. This review examines the application of multi-omics technologies, including genomics, epigenomics, transcriptomics, proteomics, and metabolomics, in relation to the diagnostic journey of rare diseases. We explore how these combined approaches enhance the detection of pathogenic genetic variants and decipher molecular mechanisms. This review highlights the groundbreaking potential of multi-omics in advancing the precision medicine paradigm for rare diseases, offering insights into future directions and clinical applications.

**Impact:**

- This review discusses using current tests and emerging technologies to diagnose pediatric rare diseases.
- We describe the next steps after inconclusive molecular testing and a structure for using multi-omics in further investigations.
- The use of multi-omics is expanding, and it is essential to incorporate it into clinical practice to enhance individualized patient care.

## Introduction

Approximately 7000 rare disorders affect over 30 million Americans, many of which lack a genetic diagnosis.^1^ Notably, around 70% of these rare genetic disorders exhibit symptoms in early childhood.^2^

These rare disorders inflict significant economic and social burden. Yang and colleagues estimated the total economic burden of 379 rare diseases in 2019 at $997 billion. This comprised 45% of direct medical costs, 44% in indirect costs due to lost productivity, 7% in non-medical costs, and 4% in uninsured healthcare costs.^3^

Beyond financial implications, rare diseases also decrease families’ quality of life. Understanding the genetic cause of the disease can improve management strategies and inform families about potential risks for future generations, prompting consideration of alternate family planning options.

Advancements in genomic technologies have enhanced diagnostic capabilities through whole exome, genome, epigenome, transcriptome, and proteome testing. Additionally, the rapid decline in sequencing costs over the past decade has allowed for large-scale studies.

This review aims to inform clinicians, trainees, and researchers about available multi-omics technologies, their application in rare genetic disorders, and their limitations in clinical settings.

### Understanding the need for a Multi-omics Approach

The complex relationship between DNA, RNA, and proteins forms the foundation of genetic information transfer. DNA serves as the genetic material, carrying instructions that are transcribed into RNA, which is crucial for protein synthesis. A comprehensive understanding of molecular processes requires integrating genomics, epigenomics (DNA), transcriptomics (RNA), proteomics (proteins), and metabolomics (metabolites). These multi-omics approaches help decipher the molecular mechanisms that connect genotype to phenotype.^4^

### Introduction to Genomics

An organism’s complete DNA set is called its genome. Nearly every cell in the human body contains a full copy of approximately 3 billion DNA base pairs that make up this genome. Genomics is the comprehensive study of the structure, function, mapping, evolution, and editing of the information encoded in our genomes.

DNA sequencing determines the exact order of bases in a DNA strand. This process is crucial for identifying genetic variations or mutations that may contribute to disease development or progression. These variations can range from small substitutions, deletions, or additions of single base pairs to large deletions of thousands of bases.

In recent decades, genomic medicine has achieved significant milestones. These include the first draft of the human genome by the National Human Genome Research Institute, the use of microarrays for detecting DNA deletions or duplications, and the emergence of whole exome and genome sequencing (WES/WGS) for identifying pathogenic variants at the nucleotide level.^5,6^ Continued advancements in genome-based research are improving diagnostics, therapeutic strategies, evidence-based clinical efficacy, and decision-making tools for patients and providers.

## WHOLE EXOME OR GENOME SEQUENCING (WES/WGS)

WES and WGS are increasingly used as first-line genetic tests for evaluating rare diseases.^7–9^ WES focuses on 2% of the genome, primarily the coding regions, while WGS covers the entire genome, including non-coding regions. WGS is superior as it analyzes an individual’s entire DNA, identifying single nucleotide variants (SNVs), copy number variants (CNVs), small insertions or deletions, and chromosomal and mitochondrial changes.^10^ WES and WGS are often performed as trio tests with the proband and biological parents, which enhances variant interpretation and diagnostic yield, thus shortening the diagnostic process.

### Utility of WES/WGS

Traditional diagnostic methods such as single gene sequencing or gene panels, often fail to identify the genetic basis of many rare diseases compared to WES and WGS.^11^ For instance, a study by the Australian National Acute Care Genomics program offered WGS to 290 families with critically ill infants suspected of genetic conditions. Results were obtained in an average of 2.9 days, with a diagnostic yield of 47%.^12,13^ Similarly, a recent meta-analysis of 23 studies in 2022 involving 1567 critically ill infants found a pooled diagnostic utility of 42% for rapid genome sequencing (*p* < 0.1).^14^ Additionally, a systematic review of 21 prospective studies with 1654 infants under one year old found a mean of 46% positive genetic test results, with 37% benefitting from rapid genome testing.^15^

WGS, which sequences both intronic and exonic regions, has shown a higher diagnostic yield compared to WES. Studies like NSIGHT1 and NICUseq have highlighted WGS’s ability to identify various genomic variants, including CNVs and SNVs.^16^ A study in NEJM indicated that WGS can identify 8% of cases that WES might miss.^17,18^

### Rapid WES/WGS

With rapid technological advancements, the turnaround time for obtaining clinical reports has significantly decreased. Tests are considered rapid if results are available in less than two weeks.^19^ This urgency is particularly critical in ICU settings, as high care costs necessitate prompt decision-making. Many studies have demonstrated the cost-effectiveness and actionable insights of rapid WES and WGS in NICU and PICU.^19–23^ From 2012 to 2021, 33 clinical studies documented the diagnostic and clinical utility of first-tier rapid whole genome sequencing in those settings.^20,21^

Turnaround time can be substantially reduced with the availability of adequate resources. For example, in 2012, disease-causing mutations in critically ill newborns were identified within 50 hours of sample collection. A recent study involving 12 patients yielded a diagnosis in as little as 5 hours for one case.^24,25^

### Role of WES/WGS in Newborn Screening

Universal newborn screening (NBS) has been pivotal for early diagnosis of various conditions, including rare inborn errors of metabolism present at birth. Certain conditions like phenylketonuria, may not exhibit symptoms initially but may lead to permanent damage if left untreated. Traditional newborn screening methods have limitations, capturing only a limited number of conditions. The emergence of WES/WGS could enhance newborn screening by enabling a comprehensive and precise diagnosis of a wider range of genetic conditions.^26^

Current NBS methods yield few false-negative results but produce many false positives results, leading to emotional and financial strain on families. For instance, a study screening 176,186 specimens by mass spectrometry (MS/MS) revealed 51 true positives, 2 false negatives, and 454 false positives. Utilizing WES/WGS could improve the specificity of existing tests and assist in diagnosing ambiguous biochemical profiles.^26,27^

Moreover, sequencing technologies could validate conditions identified through other methods, assist in prognosis and treatment decisions, and provide families with valuable information about pathogenic variants. This approach could also deepen our understanding of genotype-phenotype correlations and clarify factors contributing to false positives.

A pivotal study by Chen et al. demonstrated the advantages of genomic sequencing as a primary screening tool. The authors designed a targeted gene panel of 142 genes linked to 128 diseases, included in China’s NBS program. By enrolling nearly 30,000 newborns, they found 59 cases undetected by traditional methods but identified by gene panel sequencing, indicating that 1 in every 500 newborns could benefit from this technology. The study suggests that a genomics-based approach to newborn screening could significantly enhance detection rates.^28^

However, integrating sequencing into newborn screening presents challenges, including gaps in understanding genetic variants, complexities in data interpretation, and ethical concerns regarding data handling and consent. Addressing these challenges is crucial to obtain the benefits of genomic newborn screening while ensuring equitable access and outcomes.

### Limitations of WES/WGS

Despite their increasing accessibility, cost-effectiveness, and speed, sequencing technologies face various challenges. Results may include ‘variant of uncertain significance’ (VUS), necessitating further bioinformatic analyzes and transcriptome sequencing for accurate diagnosis. Reanalysis of genome data may reveal pathogenic variants years later, particularly as new gene discoveries emerge.^29–31^ A systematic review identified a 15% increase in diagnostic yield across 27 studies and recommended reanalysis approximately 18 months after the initial analysis.^32^

### Methods of Next Generation Sequencing (NGS) including WES/WGS

Next-generation sequencing (NGS) for WES/WGS and RNA sequencing utilizes unique sequencing chemistries and sophisticated bioinformatics to enable rapid, parallel sequencing of various DNA or RNA fragments.^33^

#### Short-read sequencing

Short-read sequencing produces reads ranging from 50 to 300 base pairs and is widely used for high-throughput genomic analyzes due to its efficiency and cost-effectiveness. Techniques such as sequencing by synthesis (SBS) and sequencing by binding (SBB) use polymerase enzymes to replicate DNA fragments to determine the nucleotide sequence of a sample.^34^

It is typically utilized for applications in WES, WGS, gene panels, and single-gene testing. However, the limited read length can pose challenges in complex genomic regions, leading to potential disparities.^35^

#### Long-read sequencing

Long-read sequencing processes DNA fragments spanning thousands of base pairs and can be categorized into ‘true’ and ‘synthetic’ long-read technologies. True long-read platforms such as PacBio and Nanopore, directly sequence long DNA strands, while synthetic long-read methods reconstruct long sequences from shorter reads.^33,35,36^

### Comparison with Optical Genome Mapping (OGM)

Optical Genome Mapping (OGM) is a non-sequencing technology by Bionano that analyzes large genomes using fluorophore-labeled DNA molecules.^34,36^ OGM can map entire chromosome arms and detect structural variations like insertions, deletions, duplications, inversions, translocations, and complex rearrangements more effectively.^36^ Several studies have shown the utility of this sequencing approach. For example, Cope and colleagues identified a mosaic deletion and inversion in CDKL5 in a patient that remained undiagnosed despite chromosomal microarray, an epilepsy panel, and exome sequencing.^37^ Additionally, a study comparing OGM and a 54-gene NGS panel in myeloid cancers found that OGM matched the performance of NGS in detecting cytogenetic abnormalities.^38^ This validates that optical mapping enhances genome assembly and gap closure when integrated with NGS.

### Role of Multi-omics when Genomic Sequencing is Inconclusive

**Fig. 1:**
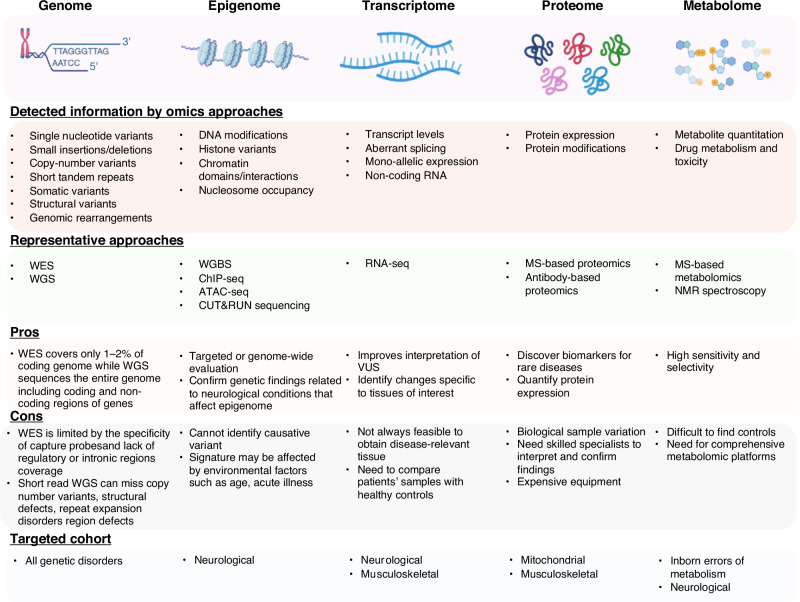
Summarizes and Compares the key multi-omics branches – genomics, epigenomics, transcriptomics, proteomics, and metabolomics. It highlights the information each detects, their advantages and disadvantages, and the types of patient cohorts they typically target.

When DNA sequencing is inconclusive, a multi-omics approach can provide a comprehensive understanding of molecular processes. This includes integrating epigenomics, transcriptomics, proteomics, and metabolomics (Fig. 1). Epigenomics explores genome-wide DNA modifications, transcriptomics analyzes RNA expression across all RNAs, proteomics uncovers protein structures, functions, interactions, and levels on a large scale, and metabolomics examines identification and quantification of metabolites in biological specimens.

## EPIGENOMICS

Epigenetic mechanisms involve chemical modifications to DNA itself or changes to proteins, such as chromatin, which interacts closely with DNA. Key modifications include DNA methylation, chromatin remodeling, histone modification, and noncoding RNA (ncRNA) -associated mechanisms.^39^ Understanding these dynamic mechanisms is crucial, as the epigenome can vary between different cell types and even among individual cells, influencing gene expression in various ways – by altering nuclear architecture, modulating transcription factor access, and directly mediating gene expression.^39^

### Types of Epigenetic Modifications

#### DNA Methylation

DNA methylation involves adding a methyl group to cytosine residues in CpG dinucleotides, primarily leading to gene silencing. This process is regulated by three types of DNA methyltransferases (DNMT): DNMT1, DNMT3A, and DNMT3B.^40^ Mutations in these enzymes are associated with various neuropathies and syndromes. For instance, *DNMT1* mutations are linked to hereditary sensory neuropathy type IE (HSANIE), mutations in *DNMT3A* are associated with overgrowth like Tatton–Brown–Rahman syndrome; while *DNMT3B* mutations are involved in immunodeficiency and intellectual disability syndromes such as immunodeficiency-centromeric instability-facial anomalies syndrome 1 (ICF 1).^41^

#### Histone Modifications

In eukaryotic cells, DNA wraps around nucleosomes formed by histone proteins, creating chromatin. The N-terminal tails of histone proteins are rich in arginine and lysine, and they undergo various post-transcriptional modifications that influence gene expression. These modifications-like methylation and acetylation – can activate or repress transcription.^39^ Dysregulation of these processes is linked to several rare diseases. For example, lysine methylation, which is crucial for gene expression, can lead to conditions such as Kabuki syndrome type 1 (*KMT2D*)and Sotos syndrome *(EHMT1)* when affected by mutations in specific genes.^42^

#### Chromatin Remodeling

Chromatin remodeling alters the accessibility of DNA to transcription factors, influencing gene expression. This process is energy-dependent, utilizing ATP hydrolysis to modify nucleosome arrangements.^43^ For instance, mutation in SMARCB1, which encodes a subunit of the SWI/SNF (switch/sucrose non-fermenting) chromatin remodeler, can reduce promoter accessibility, seen in disorders like the Coffin-Siris syndrome.^43^

#### Noncoding RNA (ncRNA)-associated Mechanisms

Recent studies indicate that up to 90% of eukaryotic genomic DNA is transcribed as ncRNAs, which regulate transcription by recruiting chromatin-remodeling complexes and facilitating epigenetic changes.^40,44^ Dysregulated ncRNAs are implicated in various conditions, including cancers and neurological disorders. For instance, deletions in the long ncRNA *CHASERR* have been associated with developmental and epileptic encephalopathy while deletions in the long ncRNA TBX2-AS1 are linked to hearing loss.^44^ Another study showed that the long ncRNA ENSG00000257522 is recurrently disrupted in individuals with microcephaly.^45^

### Types of Epigenomic Profiling Methods

#### DNA Methylation Profiling

Whole-genome bisulfite sequencing (WGBS): Combines sodium bisulfite treatment with high-throughput sequencing to map DNA methylation across the genome at single-base resolution.^46^

Methylated DNA immunoprecipitation sequencing (MeDIP-seq): Enriches and sequences methylated DNA fragments to provide a genome-wide view of DNA methylation patterns.^47^

Reduced representation bisulfite sequencing (RRBS): Targets CpG-rich regions for high-resolution DNA methylation analysis.^47^

#### Histone Modification Profiling

Chromatin immunoprecipitation sequencing (ChIP-seq): Identifies genomic regions associated with specific histone modifications or DNA-binding proteins by sequencing immunoprecipitated DNA fragments.^47^

Assay for cleavage under targets and release using nuclease (CUT&RUN): Isolates protein-DNA complexes for mapping chromatin proteins.^48^

Chromatin immunoprecipitation with exonuclease treatment (ChIP-exo): Enhances resolution of ChIP-seq by incorporating exonuclease digestion to map protein-DNA interactions precisely.^47^

#### Chromatin Accessibility Profiling

Assay for transposase-accessible chromatin using sequencing (ATAC-seq): Utilizes Tn5 transposase to insert sequencing adapters into open chromatin regions, providing insights into active regulatory elements.^49^

DNase-seq: Digests DNA at open chromatin regions using DNase 1, followed by sequencing to identify accessible chromatin regions.^47^

#### RNA Modification Profiling

N6-methyladenosine sequencing (m6A-seq): Profiles RNA modifications by immunoprecipitating m6A-modified RNA fragments followed by sequencing.^47^

#### Single-cell Epigenomic Profiling

Single-cell ATAC-seq (scATAC-seq): evaluates chromatin accessibility at the single-cell level, revealing epigenetic heterogeneity within cell populations.^49^

These methods contribute to our understanding of how epigenetic modifications regulate gene expression and cellular function, providing insights into development, disease mechanisms, and potential therapeutic targets.^49,50^

### Advancements and Limitations

Recent literature demonstrates that DNA methylation testing can diagnose up to 30% of individuals with rare neurodevelopmental conditions.^51^ This yield is comparable to the solve rates reported for chromosomal microarray (15–20%) and exome sequencing (30–40%).^51^ This suggests integrating methylation profiling into initial diagnostic assessments for specific phenotypes, such as neurodevelopmental disorders, suspected imprinting disorders, repeat expansion disorders, or a VUS in a known methylation gene.^52^

While histone modifications are imperative in neurogenesis, their roles in pediatric neurodevelopmental diseases remain poorly understood.^53,54^ Epigenetic profiles can vary significantly across different tissues, meaning blood sample findings may not always apply to the affected tissues. Additionally, the heterogeneity between phenotype and genotype further complicates the analysis of experimental results.^41^ Lastly, achieving consensus in DNA methylation analysis requires adjusting for factors such as age, sex, environmental influences, and other variables that could influence outcomes.^42^

## TRANSCRIPTOMICS

Transcriptomics studies the expression of all RNAs in a cell population, providing insights into molecular changes induced by environmental factors or pathogens. RNA sequencing has emerged as a complementary assay for rare disease diagnosis.^55^

There are four ways to analyze RNA sequencing data: expression outliers, aberrant splicing, allele-specific expression, and transcriptomic structural variants.

### Approaches to analyze RNA Sequencing Data

#### Expression Outliers

Expression outliers identify genes with unusually high or low expression levels in a sample. This analysis together with the patient’s phenotype, helps pinpoint strong candidate variants for clinical interpretation.^56^

#### Aberrant Splicing

Aberrant splicing occurs when errors disrupt the natural splicing of pre-mRNA, leading to rare diseases. Typically, noncoding sequences (introns) are removed, and certain exons are included or excluded from processed mRNA. Errors can involve exon skipping, inclusion of pseudoexons, exon extension, and intron retention, among others.^57^

#### Allele-specific Expression

Allele-specific expression (ASE) occurs when one allele is expressed at significantly higher levels than the other. In WES/WGS, single heterozygous rare variants are usually separated; however, some may exhibit ASE.^52^

#### Transcriptomic Structural Variants

Structural variants (SVs) such as translocations, duplications, inversions, and deletions can join or separate genomic regions. This can lead to gene fusions (exons from two or more distinct genes transcribed together) or altered gene functions when non-transcribed regions are included in a gene. Modifications in transcribed mRNA due to these genomic SVs are referred to as transcriptomic structural variants.^56–58^

### Advancements and Limitations

RNA sequencing can increase the diagnostic yield when WES/WGS is inconclusive. In a cohort of 113 subjects at the University of California, the diagnostic rate for WES/WGS was 31%. and incorporating RNA sequencing increased this rate to 38%.^59^ In patients with autism spectrum disorder, blood RNA sequencing identified intronic mutations and deregulated expressions in genes such as *PTEN* and *MECP2*, which had been missed by WES.^60^

As RNA sequencing technology and algorithms improve, its application in rare disease research will continue to accelerate. A comprehensive analysis of the transcriptome in each cell could uncover novel cellular and molecular components in tissues affected by rare diseases.

## PROTEOMICS

Proteins are the end product of the central dogma of molecular biology, playing a pivotal role in various cellular functions. While proteomics has historically had a lower output compared to other ‘omics technologies, it remains a valuable tool for uncovering abnormalities in protein synthesis, stability, degradation, and signaling.

Proteomics can be divided into three dimensions: expression, structural, and functional. Expression proteomics focuses on the qualitative and quantitative changes in protein composition. Structural proteomics aims on characterizing protein structures in specific cell types or organelles, while functional proteomics assesses the biological functions and mechanisms of proteins.^61^

Recent technological advancements have significantly evolved proteomics methods. Traditional techniques, such as immunohistochemistry (IHC) staining, western blotting, and enzyme-linked immunosorbent assay (ELISA), have been supplemented by high-throughput methods like tissue microarray (TMA), protein pathway array (PPA), and mass spectrometry (MS). These modern techniques not only reduce analysis time but also increase the accuracy and depth of proteome coverage.^62^

### High-Throughput Proteomic Techniques

#### Mass Spectrometry (MS)

Mass spectrometry (MS) has emerged as one of the most essential tools in proteomics. It is crucial for identifying proteins and their isoforms, as well as quantifying post-translational modifications. MS can directly detect intact proteins or specific peptide fragments, which is often challenging with traditional immunoassays. By integrating MS with various separation and pre-fractionation techniques, researchers can significantly enhance identification accuracy and yield.^62,63^

#### Protein Pathway Array (PPA)

Protein pathway array (PPA) is a gel-based high-throughput platform that uses antibody mixtures to detect antigens in protein samples extracted from biopsies or tissue. Immunofluorescence signals from antibody-antigen reactions are converted into numerical data, which provides insights into protein expression levels. This data can be further analyzed to explore biomarkers and proteomic networks.^62,63^

#### Next generation Tissue Microarrays (TMAs)

Tissue Microarrays (TMAs) consist of numerous small tissue cores from formalin-fixed paraffin-embedded (FFPE) or frozen blocks, arranged on a single histologic slide. This preparation allows for large-scale antibody-based molecular analysis of multiple samples simultaneously. TMAs are valuable for validating new biomarkers identified through PPA or MS, as well as for locating target proteins within cellular compartments such as the cell membrane, cytoplasm, or nucleus.^62,63^

### Advancements and Limitations

In the last few decades, there have been notable advancements in the field of proteomics. The UK Biobank Pharma Proteomics Project (UKB-PPP) used plasma protein signatures to develop prediction models for over 200 diseases in 41,931 patients. For 67 of these diseases, models using just 5 to 20 proteins outperformed traditional clinical models, demonstrating a median delta C-index of 0.07 (range = 0.02–0.31).^64^

Moreover, Kopajtich et al. utilized proteomics, to identify the genetic origins of rare mitochondrial diseases. Using tandem mass tag-labeled proteomics to fibroblast cell lines from 145 individuals, they identified approximately 8,000 proteins per sample, covering over 50% of Mendelian disease-associated genes.^65^ Similarly, in Spain researchers are increasingly using MS-based strategies and other proteomics methods to investigate rare inherited metabolic diseases such as methylmalonic aciduria, Fabry disease, various mucopolysaccharidoses.^61^

A significant advancement in this field is the launch of The Human Proteome Project (HPP), which seeks to map the entire human proteome using both current and emerging techniques. This initiative is expected to further enhance our understanding of rare diseases at the cellular level and open new avenues for developing therapeutic and diagnostic solutions. Recently updated guidelines for interpreting proteomic data have been published.^66^

Despite these advancements, challenges remain. Issues such as pre-analytical variables, analytical variability, and biological sample variation must be addressed to improve the performance and reproducibility of proteomics across laboratories. Additionally, the reliance on specialized operators and expensive equipment limits the routine use of proteomic tools in clinical settings. Nevertheless, as technology progresses, proteomics is expected to become a pivotal method for identifying disease biomarkers and developing more precise biochemical and immunological tests.^61^

## METABOLOMICS

Metabolomics is a comprehensive analytical approach used to study metabolites within biological specimens. Modern metabolomic technologies surpass traditional clinical chemistry techniques, enabling precise analyzes of hundreds to thousands of metabolites. Integrating metabolomics data with WES/WGS data is crucial for identifying genes associated with disease mechanisms, especially in rare and unexplained metabolic disorders in children. Unlike genes, transcripts, and proteins, metabolites have diverse physiochemical properties, necessitating multiple bioanalytical techniques for measurement, as no single technique can capture metabolite types.

### Techniques in Metabolomics

#### Nuclear Magnetic Resonance (NMR)

NMR is a spectroscopic technique that observes energetic transition of nuclear spins within a strong magnetic field. It is essential for identifying and interpreting the structures of organic molecules and metabolites, studying the dynamics of macromolecules like proteins and nucleic acids, and advancing metabolomics research. An important advantage of NMR is its reproducibility among laboratories. Standardizing procedures has become increasingly feasible, particularly for clinical applications such as the analyzing human urine, blood serum, and plasma. For instance, NMR has been used to analyze blood plasma samples from approximately 121,000 participants in the UK Biobank, resulting in a clinical chemistry panel of 249 biomarkers and ratios based on signals from lipoproteins, lipids, amino acids, and glycolysis intermediates.^67,68^

#### Mass Spectrometry (MS)

MS measures the mass-to-charge ratio (*m/z*) of ions to identify and quantify molecules in both simple and complex mixtures. This highly sensitive method can simultaneously detect and quantify thousands of metabolite features. MS is effective for the detecting, quantifying, and elucidating the structure of several hundred metabolites in a single measurement. The sensitivity and accuracy of MS depend on various experimental conditions and instrumental settings, including metabolite extraction, separation, ionization, and detection. Given the complexity of biological matrices, separating metabolites of interest before MS analysis is often necessary. Thus, analytical techniques that combine separation methods with MS, such as high-performance liquid chromatography (HPLC), gas chromatography (GS), and capillary electrophoresis (CE), have become highly effective for small-molecule analysis. However, achieving high resolution and sensitivity in a single MS detection mode is challenging, as higher sensitivity often compromises resolution, and vice versa. While MS can detect approximately 1000 metabolites, NMR can measure up to 100.^69^

### Advances and Limitations

Recent studies highlight the advancements in metabolomics. For example, Abela et al. used a combined genetic-metabolic approach to investigate early-onset epileptic encephalopathies in a cohort of 63 patients. Their untargeted metabolomic analysis identified two novel potential plasma biomarkers for Snyder-Robinson Syndrome and infantile cerebellar retinal degeneration.^70^ The development of processing tools and metabolomic databases has significantly improved the diagnosis of inherited errors of metabolism (IEM), with identified biomarkers now incorporated into newborn IEM screenings.^71^ Another study analyzed data from 19,994 patients, including plasma levels of over 900 metabolites, revealing 2599 variant-metabolite associations across 330 genomic regions.^72^

The NIH Common Fund Undiagnosed Diseases Network (UDN) developed a metabolomics program responsible for improving metabolomic methods and making them more accessible to the scientific community. This program developed the National Metabolomics Data Repository (NMDR), which houses 2397 studies from over 40 countries, containing 75,000 samples and 32,000 known metabolites. It also provides access to protocols and analytic tools that facilitate the analysis of metabolomic data through cloud computing.^73^

Biochemical changes often precede anatomical changes, suggesting that these biomarkers might predict disease conditions even in asymptomatic stages. This allows for early and effective treatment, potentially reducing complications and mortality rates. As metabolomics advances, it holds promise for enhancing our understanding of disease pathophysiology and contributing to novel treatment approaches. However, the field is still relatively new, highlighting the need for improved specialized training, reduced reliance on high-cost analytical instrumentation, and standardized operating procedures to decrease pre-analytical errors.^74^

### Challenges in Diagnosing Rare Diseases

Despite these technological advancements, diagnosing rare diseases poses several challenges. Many clinicians struggle to interpret these novel findings, creating a demand for specialists who can translate results into clinical insights. Testing is often unavailable in low-resource medical settings, necessitating referrals to specialized centers, which can lead to increased time and costs. Advanced diagnostic methods, including WES/WGS, DNA methylation, RNA sequencing, proteomics, and metabolomics require sophisticated technologies, data repositories, bioinformatic pipelines, and age/tissue-matched controls, further complicating accessibility.

Continuous funding for existing research programs dedicated to diagnosing rare and undiagnosed diseases is essential. While ongoing advancements may lead to a decline in the cost of advanced technologies, targeted sequencing, state-of-the-art computational algorithms, data-sharing platforms, and machine learning approaches will be critical in overcoming challenges related to variant detection and interpretation.

## Conclusion

Over the past two decades, gene panels, microarrays, and exome/genome sequencing have identified causal mutations in many rare disease patients, but a significant proportion remain undiagnosed. This review explores various approaches to enhance diagnostic yield and uncover the molecular mechanisms behind these diseases.

Although challenges such as cost and the need for specialists persist; there is optimism that continuous improvements will help close the diagnostic gap for undiagnosed patients; ultimately providing actionable findings. Successful implementation of multi-omics approaches in rare diseases is anticipated to revolutionize how undiagnosed patients are diagnosed and treated.

## Data Availability

This review article does not contain any primary data collection, and therefore, no new data were generated or analyzed as part of this study. The article is based solely on a comprehensive literature review of existing publications, which are cited within the text.
